# Feasibility and Preliminary Effects of Community-Based High-Intensity Functional Training for Adults with Mobility Disabilities and Overweight/Obesity: A Pilot Study

**DOI:** 10.3390/sports13100361

**Published:** 2025-10-11

**Authors:** Lyndsie M. Koon, Joseph E. Donnelly, Joseph R. Sherman, Anna M. Rice, Julianne G. Clina, John Thyfault, Reed Handlery, Kaci Handlery, Derek A. Crawford

**Affiliations:** 1 Research and Training Center on Independent Living (KU-RTCIL), Life Span Institute, University of Kansas, 1000 Sunnyside Ave., Room 4089, Lawrence, KS 66045, USA; 2 Physical Activity and Weight Management Division (DPAWM), University of Kansas Medical Center Internal Medicine, 3901 Rainbow Boulevard Mailstop 1007, Kansas City, KS 66160, USA; 3 Departments of Cell Biology and Physiology, Internal Medicine—Endocrinology, Kansas Center for Metabolism and Obesity Research, University of Kansas Diabetes Institute, Kansas City, KS 66160, USA; 4 School of Physical Therapy, Arkansas Colleges of Health Education, Fort Smith, AR 72916, USA; reed.handlery@achehealth.edu (R.H.);; 5 Department of Nutrition, Kinesiology & Health, University of Central Missouri, Warrensburg, MO 64093, USA; dcrawford@ucmo.edu

**Keywords:** high-intensity functional training, mobility disability, community-based exercise, feasibility, obesity, physical function, adaptive exercise, pilot study

## Abstract

Background: Preliminary evidence supports high-intensity functional training (HIFT) for improving various health outcomes in non-disabled adults with overweight/obesity. It remains unknown whether HIFT produces similar benefits in individuals who are overweight/obese and also have a mobility disability (e.g., spinal cord injury, multiple sclerosis)—a population disproportionately affected by obesity-related health conditions and systemic barriers to exercise. This pilot study aimed to evaluate the feasibility and preliminary effects of a 24-week HIFT intervention, delivered at community sites by certified trainers, for adults with mobility disabilities (MDs) who were overweight/obese. Methods: Twenty adults with MD and overweight/obesity (self-reported BMI 25–46 kg/m^2^) enrolled in a 24-week HIFT intervention (3 days/wk, 60 min sessions) delivered at four community-based facilities by certified trainers. Feasibility indicators included recruitment, retention, and attendance; adverse events were tracked. Effect sizes (Cohen’s *d*) were calculated for changes in obesity-related measures, physical function, work capacity, and psychological measures from baseline to post-intervention. Results: Feasibility targets were met, with a recruitment rate of 72.2%, 76.9% retention, and 80.7% attendance. Thirteen adverse events occurred. Effects on obesity-related measures ranged from negligible to moderate, with stable weight/BMI, reduced waist circumference (45% ≥ 3 cm decrease), decreased body fat, and increased lean mass. Functional outcome effects ranged from small to large and included grip strength, balance, and walking speed. Large improvements were observed for the endurance, speed, work capacity, and self-reported physical function. Conclusions: A community-based HIFT program is feasible and may improve health outcomes in adults with MD and overweight/obesity.

## 1. Introduction

Approximately 1 in 7.5 adults in the U.S. (~32.1 million Americans) have a mobility disability (MD), defined as any type of injury or illness that affects an individual’s ability to perform certain physical tasks such as self-care, housework, or ambulation [1]. This definition includes people with MD who have neurological disorders (e.g., Parkinson’s disease), health impairments (e.g., rheumatoid arthritis), congenital conditions (e.g., spina bifida), or an injury (e.g., amputation). Adults with MD are 66% more likely to be overweight or obese compared to their non-disabled peers [2,3]. This health disparity contributes to a high likelihood of health consequences including cardiovascular disease, Type 2 diabetes, chronic pain, and early mortality [2,3,4], and accounts for approximately 27% of all healthcare costs in the U.S. [1,4]. Furthermore, obesity and the accompanying conditions are linked to lower health-related quality of life, functional limitations, and reduced social participation [5,6,7,8,9]. Developing and scaling effective strategies to prevent and manage obesity-related conditions in adults with MD remains a pressing public health and clinical research priority [10,11].

Exercise is an effective strategy for improving obesity-related conditions, maintaining physical function, and promoting psychosocial health among adults with MD [12,13,14,15,16,17,18]. While rehabilitation services can temporarily address inactivity, they are often prescribed retroactively [19], and Medicaid and private insurance often limit access [20,21]. Moreover, pharmacological or surgical approaches are often prescribed more readily than exercise [22], and healthcare professionals report a lack of knowledge about where to refer patients for exercise beyond that of traditional rehabilitation [23,24]. Community-based exercise programs can support sustained engagement, but individuals with MD report numerous barriers, including inaccessible facilities and equipment, and a lack of trained staff [25,26,27,28,29].

High-Intensity Functional Training (HIFT) is a scalable, multimodal approach to exercise delivered in communities across the globe. Most prominently represented by CrossFit^®^, HIFT has been consistently ranked among the top 10 global fitness trends and now exceeds the reach of major fitness networks such as the YMCA (2650 U.S. locations) and Planet Fitness (2000 franchises) [30,31,32]. Sites operate under a decentralized infrastructure, which provides a practical platform for community-based implementation across varied geographic, organizational, and cultural contexts.

HIFT is both adaptable and accessible, and many current HIFT programs have started including people with disabilities through either formal adaptive programming or informal inclusion efforts. HIFT follows a “minimalist approach” to fitness [33], with facilities primarily housed in single-level, converted warehouse spaces—known as “boxes”—featuring accessible entryways, open workout spaces, and restrooms. The need for specialized exercise equipment is minimal, typically limited to low-cost items such as lap mats for seated participants. Existing equipment is often adapted in conventional or unconventional ways to engage individuals with disabilities, reducing both cost and accessibility barriers. HIFT programs also emphasize trainer certification through organizations like the Adaptive Training Academy (ATA), ensuring staff are equipped to deliver safe, effective, and inclusive exercise programming for people with disabilities [34].

HIFT is designed to improve physical function through multi-plane, multi-modal movements that replicate activities needed for daily living (e.g., sit-to-stand, lifting, pushing, pulling) [35,36]. These movements are especially relevant for individuals with MD, as they directly support essential tasks such as transferring, picking up an object from a seated position, or ambulating without supports. Unlike traditional programs that focus on single-joint or single-modality exercises, HIFT integrates varied compound movements that engage both aerobic and anaerobic systems, offering a more efficient and functional training stimulus that can improve physical function [35,37,38,39]. Participants can self-regulate exercise intensity, making it accessible to individuals with diverse levels of ability, fitness, and impairments [37]. Current HIFT participants with MD report improved function, independence, and general health, as well as a strong sense of community and social support through HIFT [40]. As such, HIFT has the potential to support long-term, community-based participation among people with MD and address many of the commonly reported barriers to engaging in community-based exercise.

Early findings support the feasibility and health effects of HIFT among individuals with MD [41,42] and non-disabled individuals who are overweight/obese [39,43,44]. To date, no studies have systematically evaluated its feasibility or effectiveness for improving obesity-related outcomes among adults with MD. Thus, the purpose of the current study is to evaluate the feasibility (primary) and preliminary effectiveness (secondary) of a 24-week, thrice-weekly HIFT intervention, delivered by ATA-certified trainers at community-based sites, in improving obesity-related, functional, and psychological health outcomes among adults with MD who also have overweight/obesity.

## 2. Method

**Design**. A single-group, pre-post design was used to evaluate the feasibility and preliminary effects of a 24-week HIFT program delivered across four community-based sites. Goal sample size (*n* = 25) was determined pragmatically based on available funding and feasibility of recruitment within the study timeline, rather than on formal power calculations, consistent with the pilot nature of the trial. Rolling enrollment was implemented, and written informed consent was obtained from all participants prior to participation. Procedures were approved by the institutional review board of the lead author’s institution, and the study was registered at ClinicalTrials.gov (NCT05516030). Due to the single-arm design, neither participants nor researchers were masked. Following eligibility screening, physician clearance, and informed consent, baseline surveys were administered online via REDCap [45]. A trained research team member scheduled and completed the Canadian Occupational Performance Measure (COPM) via telephone. Participants traveled to the Exercise and Human Performance Laboratory in the BEST Building on the University of Kansas Edwards Campus (Overland Park, KS, USA) to complete in-person assessments of physical health and functional performance. They were provided with detailed directions and accessible parking information in advance and were encouraged to bring assistive devices or a support person (e.g., care partner or family member) to aid with ambulation or transfers. A transfer board was available on site, and participants were permitted to bring a personal board (or other supports) if preferred to facilitate transfers onto the DXA table.

**Participants**. Participants were recruited between August 2023 and October 2024 through healthcare providers, community organizations, social media, and word-of-mouth. Eligible individuals were ≥18 years old and had a permanent mobility disability for at least one year, defined as having serious difficulty walking, climbing stairs, or lifting/carrying objects (e.g., a gallon of water). Additional inclusion criteria included a self-reported body mass index (BMI) between 25.0 and 46.0 kg/m^2^ and physician clearance to verify the MD and confirm safe participation in the program. Interested individuals completed an online eligibility questionnaire or were screened via telephone by research staff. Individuals were excluded if they reported a BMI outside the specified range, were unable to obtain physician clearance, reported sensory or intellectual/developmental disabilities, or were less than one-year post-disability onset. Participants received $30 for completing each assessment period (baseline and post-intervention), and $40 for completing a teleconference exit interview, for a total of $100. HIFT memberships were provided at no cost for the 24-week duration of the study.

**Intervention Description**. To increase accessibility, the intervention was implemented across four HIFT facilities in the greater Kansas City area. Participants were enrolled directly into ongoing, operational, adaptive HIFT classes already offered at each facility, ensuring that the intervention was delivered under real-world conditions using existing class structures and resources. Each 60 min class was capped at 10 participants and led by two Adaptive and Inclusive (AIT) certified trainers (Adaptive Training Academy), with additional support from volunteers and care partners as needed.

Before beginning the intervention, each participant completed a one-on-one orientation session with the lead trainer at their assigned intervention site. This session included a review of safety protocols, an introduction to adaptive equipment, and an assessment of baseline functional ability and assistive device needs to support individualized programming. Although participants selected a primary gym location, they were permitted to attend sessions at any of the participating facilities throughout the 24 weeks as needed. Each facility offered four adaptive classes per week at varied times (e.g., morning and/or afternoon options), and participants were encouraged to attend three classes per week based on their personal schedules. Participants could make up a missed session by attending a fourth session during the previous or subsequent week. Check-ins were conducted by research staff with participants who attended fewer than three sessions per week for two or more consecutive weeks to discuss barriers, health concerns, and support attendance by research staff. Any adverse events occurring during or following the session were documented in the training log and reported to the study coordinator according to the study’s adverse event reporting protocol.

All HIFT sessions followed a prescribed structure consisting of a warm-up, a workout of the day (WOD; ~10–25 min), and a cool-down. The lead HIFT trainer tailored the WOD for participants able to stand independently and those who remain seated during exercise, ensuring accessibility and preserving the intended stimulus of the workout (i.e., task or intensity goal). Additional individualized modifications (e.g., load, box height, range of motion) were made as needed. The WOD emphasized scalable, functional movements (e.g., lifting, pushing, pulling) targeting both aerobic and anaerobic energy systems, using formats such as AMRAPs (as many rounds as possible) in a defined period of time, EMOMs (every minute on the minute) for a defined period of time, and RFTs (rounds for time) in which a volume of work was completed as quickly as possible (see Table 1, for example, WODs). Exercises were adapted to each participant’s functional ability using equipment such as dumbbells, resistance bands, cardio machines, and bodyweight movements. Exercise intensity was guided by the Borg CR-10 scale for rating of perceived exertion (RPE) [46], a valid and reliable tool for assessing intensity during HIFT [47,48]. Participants were instructed to self-regulate intensity based on personal comfort and health status, progressing from light to moderate effort (RPE 1–3) during the initial weeks and moderate-to-vigorous effort (RPE 4–8) thereafter.

***Session adaptations and safety***. Sessions were delivered following ATA guidelines, which emphasize preserving the intended training stimulus while accommodating individual capabilities, goals, and functional limitations [34]. Exercises were adapted for both seated and standing positions. For example, squats were adapted to a dip for participants performing the exercises seated, as the dip mimics the sit-to-stand pattern of raising and lowering one’s body. For those individuals who were unable to perform a seated dip, the movement was further scaled into a seated press-down movement which allowed for the same vertical pressing stimulus. Additional adaptations accounted for range of motion, balance, and assistive device use, while safety and effectiveness were further supported through individualized scaling of exercise type, intensity, volume, and rest intervals [35]. Each participant was assigned a designated workout area equipped with fall-mitigation features, such as handrails, soft surfaces, and secure seating with safety straps when needed. Trainers provided continuous supervision during each session and adjusted movements in real time to reduce the risk of injury. Sessions concluded with structured cooldowns including breathing exercises, hydration, and stretching. Sessions were modified or paused for individual participants as needed in response to health concerns or adverse events.

***Training logs***. Trainers completed a standardized training log after each session. Logs included the original prescribed workout, the adapted version of the workout based on the participant’s ambulatory status (seated or standing), additional individualized modifications, loads used (e.g., external weights), equipment heights (e.g., boxes or platforms), and participant outcomes (i.e., rounds completed, repetitions, or total time of work). Trainers also noted any pertinent participant-reported information, such as changes in medications, fatigue, or other pertinent health-related concerns.

**Fidelity monitoring**. To monitor fidelity, trainers received weekly programming plans and completed standardized training logs documenting workout delivery, participant attendance, and any individualized adaptations. A member of the research team (e.g., PI, project coordinator) attended at least one class per week at one of the participating sites to observe program delivery and ensure adherence to safety and implementation protocols. Training logs were reviewed regularly by both the lead trainer and research staff to assess consistency with the prescribed programming and identify any deviations requiring support or corrective feedback.

## 3. Measures

**Demographic and feasibility measures**. Participant demographic characteristics (e.g., age, gender, race/ethnicity) and descriptives (e.g., disability type, assistive device use) were collected through a pre-intervention survey administered to all individuals who provided informed consent. Feasibility outcomes included recruitment rate, calculated as the proportion of eligible individuals compared to those who enrolled in the study, and retention rate, defined as the percentage of participants who completed the full 24-week intervention and post-intervention assessments. Adverse events impacting participation were documented whether they occurred during or outside of the HIFT sessions.

**Obesity-Related Measures**. For ambulatory participants, body weight in kilograms was measured twice using a portable, calibrated digital scale (Befour PS6600, Saukville, WI, USA), and standing height was assessed in duplicate using a portable stadiometer (Invicta IP0955, Leicester, UK). For participants who used wheelchairs, weight was measured in duplicate using a calibrated digital wheelchair scale (Befour MX420, Saukville, WI, USA). Net body weight was calculated by subtracting the pre-measured weight of the wheelchair from the combined participant-plus-chair weight. To ensure consistency across timepoints, digital photographs of the participant’s wheelchair were taken at baseline to verify that the same chair and accessories were used in follow-up assessments. Seated height was estimated using knee height measurements following the method outlined by Froehlich-Grobe et al. [49]. Waist circumference was measured according to the standardized protocol described by Lohman et al. [50], with three readings taken and the final value recorded as the average of the two closest measurements. Body mass index (BMI) was then calculated as participant weight in kilograms divided by height in meters squared (weight (kg)/[height (m)]^2^).

Percent body fat, lean tissue mass, and bone mineral content (BMC) were assessed using dual-energy X-ray absorptiometry (DXA; Prodigy Advance Plus, GE Healthcare, Madison, WI, USA). All participants wore hospital gowns and removed metal accessories to ensure standardized conditions. A transfer board was available for participants who required assistance transferring onto the DXA table, and trained staff provided additional support as needed. Certified technicians conducted scans following manufacturer protocols. For safety, all female participants of reproductive age completed a urine pregnancy test prior to each DXA scan. DXA data were reviewed for quality assurance, and values for total body fat and lean mass percentage, and BMC were used in analyses.

**Functional Measures**. Performance-based functional assessments were conducted at baseline and post-intervention using multiple validated performance-based tests to evaluate strength, balance, speed, and endurance. These assessments were completed in person at the University of Kansas Edwards Campus by trained assessors following standardized protocols. Self-reported physical function was assessed via telephone interviews or online surveys.

Handgrip strength was assessed using a Jamar^®^ Smart Digital hand dynamometer (Patterson Medical, Warrenville, IL, USA), with three trials conducted per hand and ~30 s of rest between each attempt. The scores (in kilograms) were averaged for dominant (self-report) and non-dominant hands. Ambulatory participants stood with feet shoulder-width apart, whereas non-ambulatory participants sat upright, both with elbows flexed at 90 degrees during assessment.

Ambulatory participants completed the Berg Balance Scale (BBS) [51], a 14-item test evaluating balance performance through functional tasks such as standing, turning, and reaching. Each item is scored on a scale from 0 to 4, with a maximum total score of 56 indicating better balance. The BBS has demonstrated strong psychometric properties in clinical populations, including high internal consistency (α > 0.90) and test–retest reliability (ICC = 0.98) among older adults and individuals with mobility disabilities [52,53]. Non-ambulatory participants completed the Function in Sitting Test (FIST) [54], a 14-item performance-based test designed to assess static, reactive, and proactive sitting balance during functional seated tasks (e.g., scooting, reaching, lifting). Each item is scored on a 0 to 4 scale, with higher scores indicating greater postural control. The FIST has shown good to excellent test–retest and interrater reliability in people with mobility disabilities [54,55,56].

Ambulatory participants completed the 10-Meter Walk Test [57] during which walking speed (m/s) was calculated by averaging two fast-paced trials. Participants were permitted to use assistive devices (e.g., cane, walker, orthotics) during testing, and the same device was used at follow-up to ensure consistency. This test has demonstrated high reliability (ICC 0.96–0.98) [58]. Non-ambulatory participants completed the 23 m Timed Forward Wheeling test conducted on a straight hard surface course as described by May et al. [59]. For these individuals, average speed (m/s) was derived by combining two fast-paced wheeling trials. Digital photographs of each participant’s wheelchair were taken at baseline to ensure consistency in equipment across all testing timepoints for speed and endurance assessments.

Endurance was assessed using the 6-Minute Walk Test (ambulatory) [60], which includes walking distance over 6 min on a 30 m loop with a 2.8 m turning radius. Results for the 6 min walk test are associated with aerobic endurance estimated by time on a treadmill to reach 85% maximum heart rate and show high test–retest reliability (ICC 0.88–0.94) [60]. Non-ambulatory participants completed the same course in their wheelchair, pushing as many meters as possible in the allotted 6 min [61].

The Canadian Occupational Performance Measure (COPM) [62] was used to evaluate participants’ perceived physical function through their ability to perform meaningful daily activities. Participants identified up to five personally significant tasks spanning areas such as self-care, productivity, and leisure. For each task, they rated both their performance (1 = cannot perform; 10 = perform extremely well) and satisfaction with performance (1 = not at all satisfied; 10 = extremely satisfied) using a 10-point scale. The COPM is well-established in rehabilitation research, demonstrating strong test–retest reliability (intraclass correlation coefficients > 0.80) [63] and sensitivity to change across clinical populations [64,65].

The Washington Group Short Set on Functioning (WGSS) [66] was used to assess disability status through self-reported functional limitations across multiple domains. Participants responded to six core items addressing difficulty with seeing, hearing, walking, cognition, self-care, and communication, as well as two additional items assessing upper body functioning (e.g., lifting/carrying) and emotional functioning (e.g., anxiety or depression) [67]. Each item was rated on a 4-point scale (1 = no difficulty; 4 = cannot do at all). This measure is widely used to identify functional disability in population-based surveys and has demonstrated strong cross-cultural validity. For descriptive purposes, responses were summarized by domain, and individuals reporting “a lot of difficulty” or “cannot do at all” in any domain were classified as having a functional limitation.

**Work Capacity**. Work capacity was assessed using three standardized tests adapted from benchmark-style workouts commonly used in HIFT programs (see Table 2) [68]. Work capacity is defined as an individual’s ability to perform mechanical work across varying modalities, intensities, and time domains [37,69]. The first work capacity test involved a 10 min AMRAP (as many rounds as possible) of rowing and dumbbell clean-and-press (standing) or ground-to-overhead (seated), with the final score representing total rounds and repetitions completed. The second test involved a 20 min AMRAP of biking, push-ups or dips, and squats (standing) or side-to-side kettlebell deadlifts (seated), measuring sustained work capacity through total rounds and repetitions. The third work capacity test was a short-duration, high-intensity sprint test—250 m row (standing) or 180 m ski (seated)—with time to completion as the outcome. The variation across each test physiologically targets a different energy system (glycolytic, oxidative, phosphagen, respectively) [70] and functional domain (e.g., lifting under fatigue, transfers, rapid-response mobility) dependent on the volume and time that the exercises were performed [35]. Testing was conducted at the HIFT facility selected by each participant by the primary trainer to ensure consistency in test delivery and scoring. Individualized loads, assistive adaptations, and equipment settings (e.g., damper level, station setup) were documented to ensure standardization across testing sessions.

**Psychological Measures**. Participants completed the Barriers to Health Activities for Daily Practice (BHADP) [71] measure at baseline and post-intervention to assess perceived obstacles to engaging in health-promoting behaviors. The BHADP is a 16-item self-report scale that asks respondents to rate the extent to which various physical, environmental, emotional, or motivational factors interfere with their ability to participate in daily health practices. Items are rated on a Likert scale, with higher scores indicating greater perceived barriers. Total scores were calculated by summing all 16 items. Global health and health-related quality of life (QOL) items from the WHOQOL-BREF [72] assessed general health, age-comparative health, satisfaction with health, and the extent to which health problems interfered with daily activities.

## 4. Data Analysis

Feasibility was assessed via recruitment, retention, attendance, and completion of outcome assessments. Participants were enrolled into a single intervention arm without randomization. The unit of analysis was the individual participant, with intervention effects assessed at the participant level. Quantitative data were analyzed in IBM SPSS Statistics (version 28, IBM Corp., Armonk, NY, USA) using descriptive statistics and effect sizes (Cohen’s *d*) to evaluate changes from baseline to post-intervention. Effect sizes values of 0.2, 0.5, and 0.7 were interpreted as small, moderate, and large effects, respectively, and were used to describe the magnitude of change and provide context for interpreting outcomes [73]. Participants with missing data at either baseline or post-intervention were excluded from the corresponding analysis. Missing data included one participant with incomplete survey responses at post-intervention, eight participants missing performance-based assessment data, and two participants who did not complete DEXA scans. Minimal clinically important differences (MCIDs) are reported when established values are available to aid in the interpretation of changes in outcome measures.

## 5. Results

**Participants**. Table 3 summarizes demographic and disability-related characteristics for the final sample (N = 20). Participants ranged in age from 18 to 79 years (Mean = 54.6 years, SD = 15.3), with an average BMI of 33.5 (SD = 7.75; range 24.1–52.1). Eligibility was based on self-reported BMI between 25 and 46 kg/m^2^; however, measured BMI at baseline indicated that one participant was below the lower limit (24.1 kg/m^2^) and three were above the upper limit (48.9–53.1 kg/m^2^). Approximately 65% of participants identified as female, and the majority identified as White/Caucasian (80%). Most participants reported using at least one assistive device (75%), and the most common disability type was categorized as an injury (35%, e.g., SCI).

**Figure 1:**
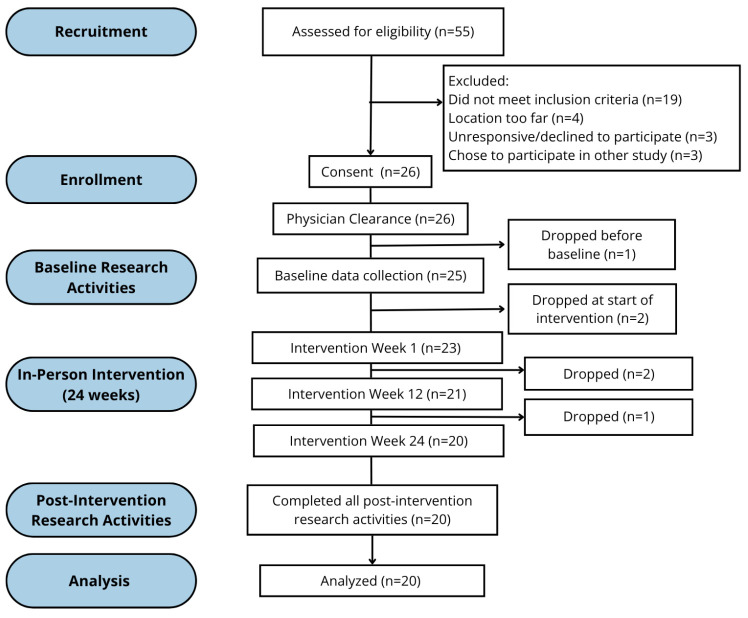
Participant flow through study, including recruitment, screening and onboarding, and retention.

**Feasibility Measures**. A total of 55 individuals were assessed for eligibility. Among 36 eligible individuals, 26 enrolled in the study, yielding a recruitment rate of 72.2%. Of the 26 participants who enrolled, 20 completed the full 24-week intervention and post-intervention assessments, resulting in a retention rate of 76.9%. One participant dropped before starting baseline assessments due to the onset of adverse health conditions, three participants dropped after the first week of the intervention began, and one participant dropped at week seven. Across all participants, the overall attendance rate was 80.7%, based on an expected maximum of 72 sessions over the 24-week intervention period. Participants attended an average of 2.42 sessions per week (SD = 0.49) across the 24-week intervention period, with weekly attendance ranging from 1.17 to 3.29 sessions. Participants were permitted to make up missed sessions by attending an additional session following an absence. Figure 1 shows participant flow through the study, beginning with recruitment, screening, intervention engagement and assessment periods. Participation following the intervention was tracked. Sixty-eight percent of participants were still actively attending HIFT classes at 8 weeks post-intervention, and 63% remained engaged at 16 weeks post-intervention. Post-intervention participation was not funded by the research study; the hosting program offered sliding-scale rates and scholarships to support continued attendance for those with financial need.

**Adverse Events**. Thirteen adverse events were recorded during the 24-week intervention, involving eight unique participants. Of these, three events (23.1%) were considered likely related to the intervention (e.g., foot pain, bicep tendonitis), while 10 events (76.9%) were unrelated, reflecting personal illness, change in medication, surgical recovery, or caregiving responsibilities. Participants sought medical care as needed throughout the intervention, and their exercise routines were adapted to support healing and a gradual return to activity based on individual needs and provider recommendations. No serious adverse events were reported. Two participants experienced surgeries unrelated to the intervention that resulted in temporary pauses in their participation. Both resumed the program following recovery and medical clearance, with appropriate modifications made to support a safe return to activity.

**Obesity-Related Outcomes**. Table 4 presents the baseline and post-intervention means, standard deviations, effect sizes, and the number of participants with complete data for each assessment. Minimal changes were observed in weight and BMI from baseline to 24 weeks (weight: *d* = −0.01; BMI: *d* = −0.05). Waist circumference showed a small to moderate reduction (*d* = −0.47). Among non-disabled adults with overweight or obesity, a reduction in waist circumference of approximately two centimeters is considered the MCID following exercise interventions [74], and reductions of 3–5 cm have been associated with reduced cardiometabolic risk [75,76]. No MCID for waist circumference has been established for adults with MD; however, evidence suggests higher waist circumference is associated with disability progression in populations with multiple sclerosis [77]. In the current study, nine participants experienced reductions ≥ 3 cm, including five with reductions ≥ 5 cm. Among participants with complete DXA data (n = 18), body fat percentage decreased (*d* = −0.36), and lean mass percentage increased (*d* = 0.38). No meaningful change was observed in bone mineral content (*d* = −0.02). While no formal MCID has been established for body fat or lean mass percentage in populations with MD, reductions in body fat percentage of 3–5% have been considered clinically beneficial [78]. In the current sample, three participants (17%) experienced ≥3% reductions in body fat percentage, while an additional four participants (22%) showed more modest reductions (~1.5–2.5%).

**Performance-Based Functional Outcomes**. Improvements were observed across multiple performance-based functional outcomes. Large effects were noted for dominant handgrip strength (*d* = 1.01), 6 min walk distance (n = 13; *d* = 1.28), 10 m walk time (n = 13; *d* = −0.95), and 23 m push time (n = 4; *d* = −1.00). Moderate effects were observed for seated balance (FIST; n = 6; *d* = 0.58) and the 6 min push distance (n = 4; *d* = 0.68). Smaller changes were observed in balance (BBS; n = 14; *d* = 0.32) and non-dominant handgrip strength (*d* = 0.23). One participant was unable to complete post-intervention walking assessments, and another was unable to complete non-dominant handgrip testing; both were excluded from respective analyses.

An increase of 5–6.5 kg in hand grip strength has been suggested as the MCID in non-disabled populations [79,80]. In the current sample, 13 participants (68%) showed improvements > 5 kg (range 5.7–8.3 kg). MCID thresholds for the 6 min walk test vary by condition. For example, individuals with SCI may require changes of approximately 45.8 m to exceed minimal detectable change [81], while among people with multiple sclerosis, an MCID of 88 m has been proposed [82]. Among adults with obesity, an MCID of ~80 m has been reported [83]. In this study, 5 of 13 ambulatory participants (38%) demonstrated improvements of ≥88 m (range: 15.3–169.1 m).

While no MCID variables have been established for the 6 min push test, the four participants all demonstrated improvements in distance pushed from baseline to post-intervention (range: 10.1–246.2 m). Although formal MCIDs for the 10 m walk test are also lacking for individuals with MD or obesity/overweight, a recent analysis in post-stroke individuals identified minimal detectable changes of 1.58 to 2.83 m/s [84]. Applying these thresholds, two of 13 participants (15%) provided by >1.6 m/s, and 1 participant (8%) improved by >2.8 s.

Seated balance improvements were observed in four of the six participants. Alzyoud et al. [85] reported MCID estimates for the FIST of 3.5 (anchor-based) and 4.8 points (distribution-based) among post-stroke participants, while another study reported an MCID of 6.5 points among individuals with various neurological types of MD (e.g., SCI, stroke) [55]. In this study, three participants exceeded these MCID thresholds, suggesting clinically meaningful improvements. For standing balance (BBS), MCID values among post-stroke individuals have been reported to range from 4 to 7 points [86,87]. In the current sample, 4 of 14 participants demonstrated improvements that met or exceeded this range.

**Self-Report Functional Outcomes**. Participants reported improvements in perceived performance and satisfaction with daily activities as measured by the COPM. Large effects were observed for both COPM performance (*d* = 1.25) and satisfaction (*d* = 1.40) scores. Scores on the WGSS-6, including upper body functioning add-ons, also improved, with a reduction in difficulty (*d* = −0.49), where lower scores indicate fewer functional limitations.

**Work Capacity Measures**. Participants demonstrated improvements across all three work capacity tests from baseline to 24 weeks. Large effects were observed for the 10 min AMRAP (*d* = 1.35) and the 20 min AMRAP (*d* = 1.39), as well as the time-based test (*d* = −0.74).

**Psychological Measures**. Improvements were observed in both psychological measures from baseline to post-intervention. A large effect was found for the BHADP scale, reflecting a reduction in perceived barriers to health-related activities (*d* = −1.36). A small, positive effect was observed for health-related quality of life (HRQOL) scores (*d* = 0.25).

## 6. Discussion

Findings from this pilot study demonstrate both the feasibility and the positive effects of a 24-week community-based HIFT program on obesity-related, functional, and psychological outcomes among adults with MD with overweight or obesity. The sample included individuals with a wide range of disability types and ages. Recruitment, retention, and attendance rates were strong (72.2%, 76.9%, and 80.7%, respectively), with relatively few adverse events. Attendance is a critical determinant of exercise dose; thus, maintaining > 80% attendance over 24 weeks supports the feasibility and acceptability of this model. These results are comparable to prior HIFT feasibility work in clinical populations, including two 25-week interventions for individuals with SCI [41] and Parkinson’s [42]. In the SCI-specific trial (n = 14; average age 60 years), attendance averaged 73% with 100% retention and no serious adverse events [41]. In the Parkinson’s trial (n = 10; average age 71.5 years), retention was 80% with attendance averaging 79% [42]. Unlike earlier studies, which were limited to single-site or therapist-led delivery, the present intervention was implemented across multiple, community-based HIFT sites and delivered by certified, adaptive HIFT trainers. An additional strength of this model is the capacity for individualized adaptations, which allowed trainers to tailor exercises to participants’ abilities, mobility needs, and assistive device use [34,40]. Prior literature suggests that this feature is particularly important for people with disabilities of differing functional capacity and progressive conditions, where exercise tolerance and physical performance may change over time [88,89]. This emphasis enhances both the inclusivity and effectiveness of adaptive HIFT for adults with MD. Furthermore, sustained engagement in the program following the intervention contrasts with many exercise trials in which participation stops abruptly at study end. This likely reflects the accessibility of the adaptive program and the strong social cohesion fostered within classes, a factor consistently linked to exercise adherence in community-based settings [25,29,90].

This study extends the evidence base by examining the impact of HIFT on obesity related outcomes among adults with MD—a population in which no prior HIFT studies have been conducted. While weight and BMI remained stable, participants demonstrated reductions in waist circumference and body fat percentage, alongside increases in lean mass percentage. These changes suggest improvements in body composition and metabolic health markers, even in the absence of weight loss. Furthermore, BMI is known to be a less accurate indicator of overweight or obesity status in people with MD, particularly those who are non-ambulatory [91,92]. Similar patterns have been found in non-disabled adults with overweight or obesity. For example, Fealy et al. [43] reported significant reductions in fat mass and preserved lean mass following a 6-week HIFT intervention in adults with overweight/obesity and type 2 diabetes, while Feito et al. [39] observed significant increases in lean mass following an 8-week HIFT intervention among sedentary adults with overweight or obesity. These results reinforce the potential of HIFT to address obesity-related health risks in adults with and without MD. Longer trials are needed to determine if prolonged exposure to HIFT would have more pronounced effects on body mass and composition in participants with MD who also have overweight/obesity.

Functional gains were also observed in the current study. For example, improvements to strength and balance were observed, as well as both walking/pushing endurance and speed improved, with the magnitude of change representing a shift from “limited community ambulation” to “full community ambulation” according to Middleton and colleagues [93]. These findings are consistent with functional gains reported in prior HIFT trials in clinical populations. In the SCI-specific intervention [41], small effects were observed for endurance via the 6-Min Arm Test (*d* = 0.20) and fast walking speed (*d* = 0.27). In the Parkinson’s trial [42], the HIFT intervention produced positive effects on endurance via the 6 min walking test (*d* = 0.57), fast walking speed (*d* = 0.42), balance (*d* = 0.93), and performance on the 5x Sit-to-Stand test (*d* = 0.55). The similarity of these patterns suggests that adaptive HIFT can elicit meaningful functional improvements across diverse neurological and mobility-impaired populations, including adults with MD who are also overweight or obese.

Our study further extends prior research by also assessing participant-perceived functional changes using COPM and the WGSS, adding a critical self-report dimension to traditional performance-based assessments. Prior literature highlights the importance of including both approaches in clinical populations, as each captures distinct and meaningful domains of functional capacity [94,95]. In our sample, the intervention produced positive changes on both measures, reflecting perceived gains in performance and satisfaction with prioritized daily activities, and in overall functional capacity. Self-report physical function has been shown to predict sustained participation in daily and community-based activities, suggesting that how participants view their function can influence long-term engagement [96]. Collectively, these functional improvements have implications beyond physical function, supporting independence in activities of daily living, reducing reliance on care partners or assistive devices, or facilitating greater participation in social and community life [17,97].

This study has several limitations. The modest sample size was pragmatically determined without a formal power calculation, consistent with the pilot nature of the study. The single-group, pre-post design and lack of masking may introduce potential biases and limit causal inference. Additional limitations include the potential for BMI misclassification, and the lack of long-term follow-up to assess the durability of effects. The sample was heterogeneous with respect to age and disability type, which may limit generalizability but also reflects the real-world diversity of individuals seeking community-based exercise options to improve various aspects of health. Future research should focus on larger, controlled trials comparing adaptive HIFT to alternative community-based exercise interventions. Such studies should incorporate implementation outcomes such as trainer fidelity, organizational readiness, and participant experience measures. Determining changes in cardiometabolic health with measures of blood lipids and glucose, and blood pressure will also be critical for future studies as individuals with MD who are at greater risk of cardiometabolic disease and sedentary lifestyle.

In conclusion, this pilot study is the first to demonstrate the feasibility and preliminary effectiveness of a community-based 24-week adaptive HIFT program, delivered by certified trainers, for adults with MD who are overweight or obese. Recruitment, retention, and attendance rates were strong across multiple sites, and the program produced meaningful improvements in obesity-related outcomes, functional performance, and perceived physical function. These gains have the potential to support greater independence, reduce reliance on assistance, and enhance community participation. Collectively, these findings reinforce adaptive HIFT as a promising, scalable, and inclusive exercise model for this underserved population, warranting further testing in larger, controlled trials with extended follow-up.

## Figures and Tables

**Table 1:** Sample HIFT workout of the day (WOD) for both standing and seated participants.

Session Type	Standing	Seated
**As many rounds as possible (AMRAP)**	10-min AMRAP: 10 Burpees; 5 squats; 10 push-ups; 5 dips	10-min AMRAP: 10 slam balls; 5 dips; 5/5 shoulder presses; 3 dips
**Rounds for Time (RFT)**	3 RFT: 20 squats; 20 sit-ups; 20 shoulder press; 20 kettlebell swings	3 RFT: 12 dips; 40 weighted twists; 20 dumbbell shoulder press; 20 dumbbell front raise
**Every minute on the minute (EMOM)**	12-min: Min 1: 18 alternating dumbbell row; Min 2: 200-m bike; Min 3: 9 deadlifts; Min 4: rest (repeat for rest of time)	12-min: Min 1: 18 alternating dumbbell row; Min 2: 200-m bike; Min 3: 10 side-to-side deadlifts; Min 4: rest (repeat for rest of time)

**Table 2:** Work Capacity Test Details.

Work Capacity	Description	Standardized Test Condition	Outcome Measured	Functional Relevance
10 min AMRAP	Perform as many rounds + repetitions as possible: 9 Calorie Row + 15 Dumbbell Clean and Press (standing); 6 Calorie Row + 15 Ground-to-Overhead Plate (seated)	Rower damper setting Load used (DBs or plate) Rower serial #	Total rounds and repetitions completed in 10 min	Lifting/carrying under fatigue Multi-joint movement integration
20 min AMRAP	Perform as many rounds + repetitions as possible: 5 Calorie Bike + 7 Push-ups + 9 Box Squats (standing); 2 Dips + 3 Calorie Arms-Only Bike + 8 Side-to-Side Deadlifts with Kettlebell (seated)	Push-up/dip bar height Box height/ Kettlebell weight Bike serial # Station setup for transition time	Total rounds and repetitions completed in 20 min	Transfers, toileting, lap-floor reach Total-body endurance in functional tasks
For Time	Complete: 250 m Row (standing); 180 m Ski (seated)	Damper setting Row/SkiErg serial # Consistent warm-up protocol	Time to completion	High-output, rapid work tasks (e.g., emergency transfers, sprint-carry, quick response in daily activities)

**Note.** AMRAP = As Many Rounds As Possible; DB = Dumbbell; # = serial number.

**Table 3:** Participant demographics and descriptives.

Group	N = 20		
Variable	Category	*n*	%
Sex	Female	13	65.0%
	Male	7	35.0%
Age	18–35	3	15%
	36–54	7	35%
	55–79	10	50%
BMI *	24.1–33.4	14	70%
	33.5–42.7	2	10%
	42.8–54.1	4	20%
Education	Graduate degree	9	45%
	College completed	5	25%
	1–3 years of college	4	20%
	High School completed	1	5%
	Other	1	5%
Race	White	16	80%
	Black/African American	3	15%
	Do not wish to answer	1	5%
Disability Type	Neurological		
	(Ataxia, Multiple Sclerosis)	6	30%
	Injury		
	(SCI/D)	7	35%
	Health Impairment		
	(Stroke; GPA)	6	30%
Assistive Devices	Utilizes an assistive device	15	75%
	(e.g., cane, walker, manual wheelchair, orthotic devices)		
	Does not utilize assistive devices	5	25%

**Note:** BMI * = calculated body mass index. SCI/D = Spinal cord injury or disease; GPA = Granulomatosis with polyangiitis (formerly Wegener’s granulomatosis).

**Table 4:** Outcome means, standard deviations at intervention baseline and endpoint plus mean change, standard deviation, and effect size (Cohen’s d).

Measure	N	Baseline Mean (SD)	Endpoint Mean (SD)	Mean Change (Pooled SD)	Effect Size (Cohen’s d)
***Obesity-Related Outcomes***					
Weight (kg) ^a^	20	97.64 (22.72)	97.46 (23.24)	−0.18 (22.98)	−0.01
Body Mass Index (weight (kg)/[height (m)]^2^) ^b^	20	34.39 (8.77)	34.32 (8.82)	−0.08 (1.40)	−0.05
Waist Circumference (cm) ^c^	20	106.29 (15.54)	104.65 (16.86)	−1.64 (3.47)	−0.47
Body Fat % ^d^	18	42.8% (9.8%)	41.4% (9.3%)	−1.35% (0.03%)	−0.36
Lean % ^d^	18	57.1% (9.8%)	58.6% (9.3%)	+1.43% (3.76%)	0.38
Bone Mineral Content (g) ^d^	18	2814.89 (665.85)	2813.11 (712.92)	−1.78 (110.90)	−0.02
***Performance-based Functional Health Outcomes***					
Grip Strength (Dominant) ^e^	20	26.81 (11.98)	29.56 (12.88)	2.25 (2.72)	1.01 *
Grip Strength (Non-Dominant) ^e^	19	23.43 (12.29)	24.35 (12.35)	0.91 (4.05)	0.23
Berg Balance Scale (BBS) ^f^	14	49.9 (4.4)	51.2 (4.5)	1.36 (4.22)	0.32
Function in Sitting Test (FIST) ^f^	6	38.2 (10.6)	42.0 (6.4)	3.8 (6.6)	0.58
10-Meter Walk Time (m/s) ^g^	13	7.7 (2.8)	6.9 (2.7)	−0.71 (0.74)	−0.95 *
23-Meter Push Time (m/s) ^g^	4	9.8 (1.8)	9.0 (1.4)	−0.83 (0.83)	−1.00 *
6-Minute Walk Distance (m) ^h^	13	348.7 (132.3)	422.3 (141.7)	73.61 (57.63)	1.28 *
6-Minute Push Distance (m) ^h^	4	462.3 (37.9)	539.6 (105.5)	77.4 (24.1)	0.68
***Self-Report Functional Health Outcomes***					
COPM Performance ^i^	20	5.09 (1.41)	6.58 (1.02)	1.49 (1.19)	1.25 *
COPM Satisfaction ^i^	20	4.36 (1.85)	6.46 (1.38)	2.12 (1.51)	1.40 *
WGSS-6 + Upper Body Functioning Add-ons ^j^	20	12.45 (2.09)	11.7 (1.66)	−0.85 (1.73)	−0.49
***Work Capacity***					
Work Capacity (10 min AMRAP; rounds.repetitions) ^k^	18	3.69 (0.73)	4.77 (1.07)	1.08 (0.80)	1.35 *
Work Capacity (20 min AMRAP; rounds.repetitions) ^k^	19	7.68 (1.90)	11.20 (3.46)	3.41 (2.45)	1.39 *
Work Capacity (s)	19	81.10 (27.61)	64.25 (14.02)	−15.91 (21.44)	−0.74
***Psychological Outcomes***					
Quality of Life (WHOQOL-BREF) ^l^	20	11.75 (1.92)	12.15 (2.30)	0.40 (1.60)	0.25
Barriers (BHADP) ^m^	19	26.53 (4.73)	24.84 (5.29)	−3.00 (2.21)	−1.36 *

**Note.** * *Indicates large effects (Cohen’s d ≥ 0.70). All values represent group means and standard deviations (SD).* ^a^ Weight measured in kilograms (kg) to the nearest 0.1 kg. ^b^ Body Mass Index (BMI) calculated as weight (kg) ÷ height (m^2^). ^c^ Waist circumference measured to the nearest 0.1 cm. ^d^ Body composition assessed via dual-energy X-ray absorptiometry (DEXA); values reported as percent fat mass (fat %), percent lean mass (lean %) and bone mineral content (BMC) in grams. ^e^ Grip strength measured in kilograms using a standardized hand-held dynamometer. ^f^ Berg Balance Scale (BBS) and Function in Sitting Test (FIST) are standardized clinical assessments of balance. Both consist of 14 items scored 0–4, with total scores ranging 0–56; higher scores indicate better balance/function. ^g^ 10-Meter Walk and 23-Meter Push: Reported as average speed in m/sec (lower times = faster performance). ^h^ 6-Minute Walk/Push Test (6MWT): Ambulatory participants completed the test without assistive devices (although use was permitted if needed). Wheelchair users completed the test in their own manual wheelchair. ^i^ Canadian Occupational Performance Measure (COPM) Performance and Satisfaction scores range from 1–10; higher scores indicate greater perceived performance/satisfaction. ^j^ Washington Group Short Set on Functioning—6-item version with Upper Body Functioning add-ons (WGSS-6+UBF): Scores range from 0–18; lower scores indicate fewer functional difficulties. ^k^ AMRAP: as many rounds as possible. ^l^ WHOQOL-BREF total scores range from 4–20; higher scores reflect better perceived quality of life. ^m^ Barriers to Health-promoting Activities for Disabled Persons (BHADP) total scores range from 16–64; higher scores reflect more perceived barriers to health behaviors.

## Data Availability

The original contributions presented in this study are included in the article. Further inquiries can be directed to the corresponding author.

## Abbreviations

The following abbreviations are used in this manuscript:

AIT	Adaptive and Inclusive Trainer
AMRAP	As Many Rounds As Possible
ATA	Adaptive Training Academy
BBS	Berg Balance Scale
BHADP	Barriers to Health Activities for Daily Practice
BMC	Bone Mineral Content
BMI	Body Mass Index
COPM	Canadian Occupational Performance Measure
RPE	Rate of Perceived Exertion
DB	Dumbbell
DXA	Dual-Energy X-ray Absorptiometry
EMOM	Every Minute on the Minute
FIST	Function in Sitting Test
HIFT	High-Intensity Functional Training
QOL	Quality of Life
ICC	Intraclass Correlation Coefficient
MCID	Minimal Clinically Important Difference
MD	Mobility Disabilities
RFT	Rounds for Time
SCI	Spinal Cord Injury
WHOQOL-BREF	World Health Organization Quality of Life-BREF
WGSS	Washington Group Short Set on Functioning
WOD	Workout of the Day
